# The Transmission of SARS-CoV-2 from COVID-19-Diagnosed People to Their Pet Dogs and Cats in a Multi-Year Surveillance Project

**DOI:** 10.3390/v16071157

**Published:** 2024-07-18

**Authors:** Anne K. Kimmerlein, Talon S. McKee, Philip J. Bergman, Irina Sokolchik, Christian M. Leutenegger

**Affiliations:** 1 VCA Animal Hospitals, Los Angeles, CA 90064, USA; 2 VCA Clinical Studies, Los Angeles, CA 90064, USA; talon.mckee@vca.com (T.S.M.); philip.bergman@vca.com (P.J.B.); 3 Immunology R&D, Antech Diagnostics, Brownsburg, IN 46112, USA; irina.sokolchik@antechmail.com; 4 Research and Development, Antech Diagnostics, Fountain Valley, CA 92708, USA; christian.leutenegger@antechmail.com

**Keywords:** SARS-CoV-2, COVID-19, pets, disease surveillance, public health, One Health, zoonoses, viruses

## Abstract

Recent emerging zoonotic disease outbreaks, such as that of SARS-CoV-2, have demonstrated the need for wider companion animal disease surveillance. We tested 1000 dogs and cats belonging to employees of a US veterinary hospital network that were exposed to human COVID-19 cases in the household between 1 January 2020 and 10 March 2022 for SARS-CoV-2 and surveyed their owners about clinical signs and risk factors. The seropositivity was 33% for 747 dogs and 27% for 253 cats. Pet seropositivity correlated with the US human case rates over time, exhibiting peaks corresponding with the major COVID-19 surges. Antibodies persisted longer than previously documented (828 days in dogs; 650 days in cats). Increasing age and duration of proximity to infected people were associated with increased seropositivity in dogs but not cats. Cats were more likely to have clinical signs, but an association between seropositivity and the presence of clinical signs was not found in either species.

## 1. Introduction

From reducing the risk of heart attacks to alleviating loneliness, pet ownership benefits individuals and contributes to healthy communities [1,2,3,4]. Approximately 66% (86.9 million) of US households included at least one pet in 2022 [5]. Surveys of pet owners suggest that close contact with pets, including cuddling, sharing food, and sleeping in the same bed, is common [6,7], and 83% of survey respondents said they spend most or a large part of the day with their pets [8]. Moreover, pets are becoming increasingly common in public spaces and workplaces [9,10,11]. Despite their many benefits, interaction with pets comes with the risk of zoonotic disease [6,12]. Yet veterinary disease surveillance has traditionally focused on food safety [13,14,15], with limited companion animal zoonotic disease surveillance [16,17,18]. Recent emerging infectious disease outbreaks, such as that of SARS-CoV-2, have demonstrated the need for rapid disease detection and response at the intersection of human and animal health [19]. Yet One Health reporting and surveillance systems remain disjointed, and companion animal disease detection, monitoring, and response are not standard components of most public health or animal health programs [14].

In response to the COVID-19 pandemic, multiple academic studies were launched to investigate the potential role of pets in SARS-CoV-2 transmission [7,20,21,22,23]. These studies were typically limited to specific geographic locations and narrow time periods. Wider testing using remnant samples from veterinary diagnostic laboratories lacked context without associated patient history [24,25,26]. We present a novel approach to large-scale, nationwide companion animal disease surveillance. Pets living with employees of a veterinary hospital network in the United States were tested for SARS-CoV-2 via collaboration with a commercial veterinary diagnostic laboratory. In this study, we determined SARS-CoV-2 seroprevalence in dogs and cats exposed to human cases of COVID-19 over a two-year time period and across the United States. Additionally, we explored the antibody response patterns in dogs and cats and identified risk factors for household reverse zoonosis of SARS-CoV-2 from people to their pets.

## 2. Materials and Methods

### 2.1. Survey

All VCA Animal Hospitals employees located in the United States were invited to enroll their dogs or cats in the study via an online survey link sent to their company email address and via announcements on the company internal social media platform. In order to qualify for the study, at least one person in the employee’s household had to have been diagnosed with COVID-19 at least 2 weeks prior to study enrollment. Households with current COVID-19 infections were asked to wait 2 weeks past the resolution of clinical signs to enroll in the study. Participants were asked to report the date on which a member of their household tested positive for COVID-19 and how many people in their household had COVID-19 and provide basic health and lifestyle information about their pet from the time at which COVID-19 was present in the household. Participants were not asked to provide medical information about themselves or other people in their household. Participants were allowed to enroll as many pets from their household as they wished, but each pet could only be enrolled and tested once.

### 2.2. Sample Collection

The study samples were collected from 23 September 2021 to 11 May 2022. Upon completion of the survey, participants were asked to submit a single whole blood sample for each enrolled pet. Blood was collected in an EDTA Vacutainer tube at the VCA hospital of the participant’s choice and dispatched to Antech Diagnostics via the hospital’s routine laboratory courier service. Samples were accepted from the date of pet enrollment to the end of the study period. Ethical approval for this study was granted by the VCA Clinical Studies Institutional Review Board (VCSIRB-AN2A), and written informed consent was collected from each person participating in the study for each pet enrolled.

### 2.3. ELISA

SARS-CoV-2-specific antibodies in cats and dogs were measured using enzyme-linked immunosorbent assay (ELISA). Plates were coated with SARS-CoV-2 recombinant nucleocapsid protein (2.2 µg/mL, MP Biomedicals, Irvine, CA, USA) or recombinant spike protein (1 µg/mL, Sino Biological, Chesterbrook, PA, USA). Horseradish peroxidase-conjugated goat anti-cat and goat anti-dog IgM and IgG (Sigma-Aldrich, St. Louis, MO, USA) were used to detect bound antibodies. The ELISA plates were coated overnight at 4 °C and then blocked using 5% non-fat powdered milk (1 μg/mL, 100 μL per well) in 1× PBS for 2 h at 37 °C. Serum samples at a dilution of 1:200 were incubated for 30 min on a rocking platform and then washed 5 times with PBS containing 0.05% Tween-20 (Sigma-Aldrich, St. Louis, MO, USA). Conjugated antibody was added at 1:100 and 1:400 (anti-dog IgG or IgM, respectively) or 1:80 (anti-cat IgG and IgM) and incubated for 30 min at 18–25 °C. After 5 wash steps, room-temperature TMB Substrate (Sigma-Aldrich, St. Louis, MO, USA) was added and incubated for 15 min at 18–25 °C. Color reactions were stopped using 0.33 M sulfuric acid (H2SO4) and the OD determined at 450 nm. Sera were considered antibody-positive when the mean OD of the replicates was 2 standard deviations above the positive control. Serum samples from two PCR-confirmed SARS-CoV-2-infected cats were used to validate the ELISA protocol, and specific feline and canine reference serum samples were used as positive controls in the screening process.

### 2.4. Statistical Analysis

The data analysis was performed using R Statistical Software (v4.1.1; R Core Team 2021). Statistical differences were determined at the 5% level of significance. The proportions of anti-spike and anti-nucleocapsid antibodies were compared via a clustered Chi-Square goodness-of-fit test [27]. Generalized mixed effects logistic regression (GLMER), with household included as a random effect, was used to evaluate the univariate associations between seropositivity and individual risk factors, including animal signalment, geographical location, lifestyle factors, and household exposure factors. Tukey’s multiple comparisons of means was used to further characterize the significant differences identified for multilevel factors. Factors with a significant association (*p* < 0.05) in the univariate analysis were included in the multivariate GLMER modeling. All possible interactions between the predictor variables were explored and retained in the final model if statistically significant. Final multivariate models were selected via a manual backwards elimination procedure using the Likelihood Ratio Test and scientific best judgement. GLMER was also used to evaluate the frequencies of antibodies and binding sites based on the time between COVID-19 exposure and testing. Daily national human COVID-19 case counts were downloaded from the Johns Hopkins University Coronavirus Resource Center [28] and aggregated into monthly counts. A time series analysis using a cross-correlation function was performed to determine whether there was a relationship between national human COVID-19 case reporting data and the dog and cat study cases by date of exposure.

## 3. Results

**Figure 1:**
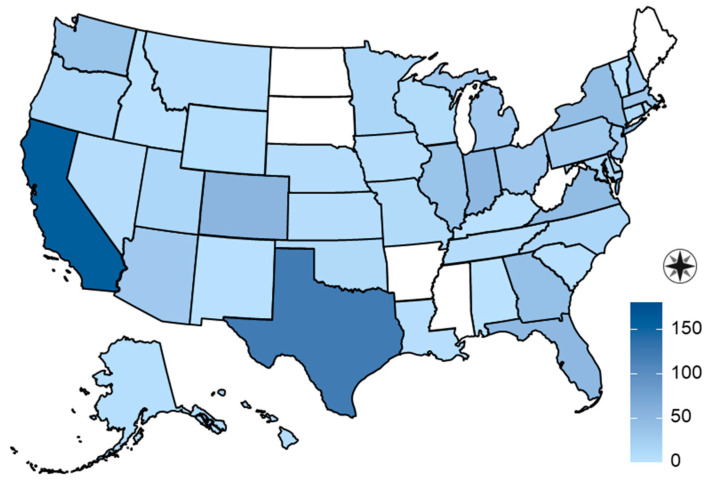
Geographic distribution of 747 dogs and 253 cats in a study of household transmission of SARS-CoV-2 from people to pets.

A total of 1299 surveys were completed, with 1233 representing unique, eligible pets. Of those, 1000 blood samples were received from 747 dogs and 253 cats. The samples represented 681 households from 44 states and Washington, D.C. (Figure 1). Two-hundred and twenty-one (32%) households submitted a sample for more than one pet. The median number of samples per household was one (range: 1–8). The dogs were a median of 6 years of age (range: 5 months–18 years), and the cats were a median of 6 years of age (range: 6 months–20 years). The dogs included 370 (50%) females and 377 (50%) males, while 664 (89%) were spayed or neutered. Cats included 113 (45%) females and 140 (55%) males, while 235 (93%) were spayed or neutered.

### 3.1. ELISA

The overall seroprevalence was 33% for dogs and 27% for cats. Both dogs and cats had significantly (*p* < 0.001) higher frequencies of IgG against the spike protein (dogs: 27%; cats: 22%) compared to that against the nucleocapsid protein (dogs: 11%; cats: 13%). However, neither dogs nor cats had significantly different (dogs: *p* = 0.25; cats: *p* = 0.16) frequencies of IgM against the spike protein (dogs: 6%; cats: 0.8%) versus that against the nucleocapsid protein (dogs: 7%; cats: 0%). When anti-spike and anti-nucleocapsid IgG antibodies were combined, 225 (30%) dogs and 67 (27%) cats were seropositive for IgG. When anti-spike and anti-nucleocapsid IgM antibodies were combined, 70 (9%) dogs were seropositive for IgM. Only two cats (0.8%) had antibodies against the IgM spike protein, and none had antibodies against the IgM nucleocapsid protein.

**Figure 2:**
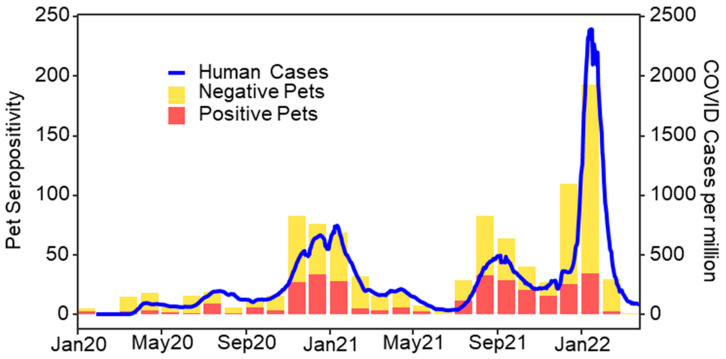
Monthly human COVID-19 cases per million people and SARS-CoV-2 seroprevalence for 747 dogs and 253 cats with household exposure to human COVID-19 cases between 1 January 2020 and 10 March 2022, USA.

The owner-reported dog and cat SARS-CoV-2 exposure dates ranged from 1 January 2020 to 10 March 2022. The study’s seropositivity rates showed a distinct, multi-waved temporal pattern similar to that for US human case rates, with corresponding peaks in Winter 2020–2021, Fall 2021, and Winter 2021–2022 (Figure 2). There was a statistically significant (*p* < 0.001) cross-correlation between the monthly national human COVID-19 case counts and the seropositive dogs and cats in the study, despite a noticeably smaller peak in pet seropositivity compared to human cases in Winter 2021–2022 (Figure 2). No significant difference in seropositivity was found between US Census Bureau Regions [29] for dogs (*p* = 0.51) or cats (*p* = 0.86).

The median time between owner-reported exposure to a person with COVID-19 in the household and testing was 116 days (range: 11–828 days) for dogs and 87 days (range: 16–682 days) for cats. In dogs, IgG was detected between 16 and 828 days and IgM was detected between 17 and 812 days after exposure to COVID-19 in the household. In cats, IgG was detected at 19 to 650 days and IgM was detected at 38 to 81 days after exposure to COVID-19 in the household. There was no significant relationship between the type of antibody present and the number of days between exposure to COVID-19 in the household and testing in either dogs (*p* = 0.30) or cats (*p* = 0.43). Dogs developed IgG against nucleocapsid proteins significantly earlier (median: 72 days; range: 16–551 days) than IgG against spike proteins (median: 177 days; range: 17–828 days; *p* = 0.008). In cats, there was no significant difference (*p* = 0.97) in the time from COVID-19 exposure to testing between anti-nucleocapsid IgG (median: 106 days; range: 19–589 days) and anti-spike IgG (median: 115 days; range: 29–650 days). There was no significant difference (*p* = 0.63) in time from exposure to testing between anti-nucleocapsid (median: 93 days; range: 18–812 days) and anti-spike IgM (median: 97 days; range: 17–551 days) in dogs.

### 3.2. Lifestyle Factors

**Figure 3:**
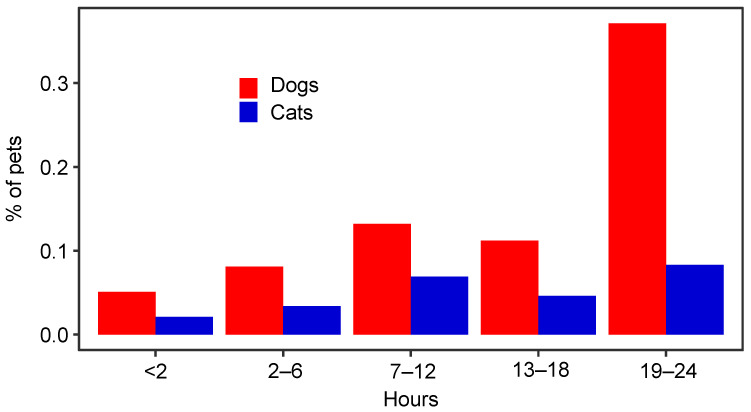
Time that 747 dogs and 253 cats spent in the room with a COVID-19-diagnosed person in a study of household transmission of SARS-CoV-2 from people to pets, USA.

The majority of the dogs (81%), but few cats (13%), engaged in activities outside the home while a person in the household had COVID-19. The most common activities for dogs during this time were on-leash walks (50%) and spending time in a fenced yard or tied up (46%). Fewer dogs visited an off-leash or dog park (5%) or went to a groomer or boarding facility (2%). The majority of the cats (79%) were described as indoor-only, and none were described as mainly or exclusively outdoor cats. Few cats (10%) were reported to have spent unsupervised time outdoors while there was COVID-19 in the household. Seventy-two dogs (10%) and eight cats (3%) were taken to a veterinary clinic during the time that COVID-19 was present in the household. Nearly all the dogs (90%) and cats (95%) sat on the lap of, and most of the dogs (71%) and cats (87%) slept in or on the bed of, a person with COVID-19. The majority of the dogs (85%) and roughly half of the cats (53%) licked the face or hands of or were kissed by (72% of dogs; 66% of cats) an infected person. Most of the dogs (65%) and roughly half of the cats (51%) spent over 12 h per day in the same room as a person with COVID-19 (Figure 3).

For the analysis of all the risk factors, seropositivity was defined as testing positive for one or more antibodies against SARS-CoV-2. For dogs, the odds of seropositivity increased by 5% with every one-year increase in age (*p* = 0.04; Table 1), but age was not associated with seropositivity in cats (*p* = 0.58). Dogs who spent fewer than 2 h in the same room as an infected person had 76% lower odds of being seropositive (*p* = 0.004; Table 1), but differences in seropositivity were not found between the other time periods evaluated. No lifestyle factors were significantly associated with seropositivity in cats (Table 2). The number of people who were infected with COVID-19 in the household did not significantly affect seropositivity in dogs (*p* = 0.14) or cats (*p* = 0.96).

### 3.3. Clinical Signs

The owners reported at least one respiratory, gastrointestinal, or systemic clinical sign, such as decreased appetite or energy, in 95 (13%) dogs and 57 (23%) cats at the time that a human member of the household had COVID-19. Respiratory signs (coughing, sneezing, difficulty breathing) were the most frequently reported, in 53 (7%) dogs and 45 (18%) cats. The owners reported significantly more seropositive cats experienced sneezing (12%) and decreased appetite (10%) than did seropositive dogs (4% sneezing, *p* = 0.02; 3% decreased appetite, *p* = 0.01) at the time of household COVID-19 exposure. However, no significant associations were found between seropositivity and any of the owner-reported clinical signs at the time of household COVID-19 exposure for either species (Table 3).

## 4. Discussion

This study presents a novel approach to large-scale companion animal zoonotic disease surveillance, whereby pet dogs and cats living with employees of a large US veterinary hospital network were tested for SARS-CoV-2 via collaboration with a commercial veterinary diagnostic laboratory. While several studies at academic institutions have investigated the role of pets in SARS-CoV-2 transmission, these were conducted in restricted geographic locations and specific timeframes during the pandemic [7,20,22,23,30,31,32]. This study is the first to describe the epidemiology of SARS-CoV-2 in pets across the United States and over an extended time period. The pets in this study were naturally exposed to human COVID-19 cases at home from the start of the pandemic in early 2020 through to Spring 2022, encompassing a greater than two-year time period. SARS-CoV-2 seropositivity in study pets was significantly correlated with US human case rates over time and exhibited obvious peaks in seropositivity for pet exposure corresponding with the three main human case surges in the US, including those caused predominantly by the Delta and Omicron variants [33]. While the Omicron variant has been shown to have greater transmissibility among humans [34], beagle dogs experimentally infected with the Omicron variant had lower viral loads and a shorter shedding time than dogs infected with the Delta variant [35]. This difference may help explain the noticeably smaller peak in pet seropositivity compared to human case rates during the Winter 2021–2022 Omicron surge.

Dog and cat SARS-CoV-2 seroprevalence in this study was centered within the ranges presented by previous studies evaluating pets exposed to human COVID-19 cases in the home (11–40% in dogs, 21–44% in cats) [7,20,21,22,23,36,37,38]. The variation in seroprevalence found among other studies may have been a consequence of the limited geography and specific date ranges of those studies, which were smoothed by the large geography and the long exposure period in this study.

The humoral response to SARS-CoV-2 infection has been well studied in people [39,40,41], but animal SARS-CoV-2 studies have prioritized antibodies as markers of previous infection, and the techniques for antibody detection, quantification, and reporting have varied [7,20,22,23,42]. In humans, antibodies against SARS-CoV-2 follow the expected patterns: IgM is detected 4 days after symptom onset but declines after about 20–30 days; IgG is detected within 7 days of symptom onset, maintains high levels for 3–6 months, and declines roughly 100 days after the onset of clinical signs [41,43]. In experimentally infected cats, IgM was detected at low levels relative to IgG at 7 days post inoculation and was undetectable by day 28, while IgG levels progressively increased between 7 and 28 days post inoculation [44]. Our study found higher frequencies of IgG than IgM antibodies in both dogs and cats. Immunoglobulin antibodies persisted much longer than the previously documented 2-10 months in dogs and cats after exposure to people with COVID-19 in the household [23,44,45]. In dogs, IgG and IgM were detected more than 2 years after reported exposure to COVID-19 in the household. In cats, IgG was detected almost 2 years after exposure, but only two cats were seropositive for IgM, both under 3 months after exposure to COVID-19 in the household. The less robust antibody response in cats may help explain the increased susceptibility to infection and higher proportion of clinical signs seen in cats compared to dogs in this study and others [20,42,46]. The sustained presence of antibodies against SARS-CoV-2 may help explain the relatively few and mild clinical cases of SARS-CoV-2 in dogs and cats [42,44,47]. However, one limitation of this study is that the survey only asked for the date of the pets’ first known exposure to COVID-19 in the household. It is possible that unreported or unrecognized re-exposure of pets occurring between the study enrollment and sample submission artificially extended the length of time for which antibodies were detected after exposure.

Previous risk factor analyses for SARS-CoV-2 infection in pets have provided variable results, due, in part, to small sample sizes [7,20,42]. As in other studies, close human–animal contact was common between the studied pets and people in households with COVID-19 [7,20]. We found an association between increasing age and seropositivity in dogs, which had not been previously identified. Fewer than 2 h spent in the same room as an infected person was associated with reduced seropositivity in dogs, but specific types of human–animal contact were not any more or less associated with pet seropositivity. These findings may indicate that host-specific factors play a greater role than household factors in dog and cat SARS-CoV-2 susceptibility, and only the strictest precautions, such as complete isolation, are helpful in reducing human-to-pet SARS-CoV-2 transmission. Unfortunately, we did not structure our survey in a way that facilitated differentiation between minimal time with and strict isolation from infected people.

Cats were more likely to have clinical signs than dogs, but unlike previous studies [7,20,42], we did not find an association between seropositivity and the presence of clinical signs. Our study allowed for prolonged periods between cases of COVID-19 in the household and survey completion, which may have affected the participants’ ability to remember their pets’ clinical signs. Additionally, all the participants in this study were employed in the veterinary field, which may have led to differing standards regarding what were considered noteworthy or concerning clinical signs compared to those of the general public.

Pets hold a special place in our society and are often closely integrated into our households and daily routines. However, these close relationships put both humans and animals at risk of zoonotic disease transmission. The necessity to rapidly investigate the role of pets in the SARS-CoV-2 pandemic emphasized the need for more comprehensive and agile companion animal zoonotic disease surveillance. This first-of-its-kind clinical study brought together hundreds of VCA employees from across the US to help address a critical knowledge gap in an emerging public health pandemic. This study demonstrates the power of cooperation between commercial laboratories and large veterinary hospital networks to help meet the ever-changing and growing needs of the veterinary community.

## Figures and Tables

**Table 1:** Results of regression model analysis evaluating lifestyle risk factors for SARS-CoV-2 seropositivity in 747 U.S. dogs with household exposure to human COVID-19 cases between 1 January 2020 and 10 March 2022 ^1^.

Variable	β	SE	OR (95% CI)	*p* Value
Age (years)	0.044	0.022	1.05 (1.01, 1.09)	0.04
<2 h in room with infected person	−1.425	0.501	0.24 (0.09, 0.64)	0.004
Slept in or on bed of infected person	0.215	0.196	1.24 (0.85, 1.82)	0.27

^1^ Seropositivity was defined by IgG or IgM against viral spike or nucleocapsid protein. The model was adjusted for household clustering.

**Table 2:** Association between SARS-CoV-2 seropositivity and lifestyle risk factors in U.S. dogs and cats with household exposure to human COVID-19 cases between 1 January 2020 and 10 March 2022 ^1^.

Characteristic	Dogs			Cats		
	Seronegative N = 500	Seropositive N = 247	*p* Value	Seronegative N = 185	Seropositive N = 68	*p* Value
Age, y, median (range)	6 (0.4, 18)	7 (0.5, 16)	0.03	6 (0.5, 20)	5 (0.8, 16)	0.58
Feline lifestyle						0.53
Indoor exclusively				143 (77)	57 (84)	
Mainly indoor with some outdoor access				30 (16)	8 (12)	
Large amounts of time indoors and outdoors				12 (7)	3 (4)	
Went on leashed walks	253 (51)	122 (49)	0.77			
Went to an off-leash or dog park	26 (5)	13 (5)	0.28			
Spent unsupervised time outdoors ^†^	219 (44)	123 (50)	0.16	19 (10)	8 (12)	0.75
Visited a veterinary clinic	56 (11)	16 (6)	0.06	3 (2)	5 (7)	0.05
Went to a groomer or boarding facility	28 (6)	7 (3)	0.11	0 (0)	0 (0)	
Slept in or on the bed of an infected person	341 (68)	189 (77)	0.03	161 (87)	59 (87)	0.89
Licked the face or hands of an infected person	423 (85)	215 (87)	0.43	93 (50)	41 (60)	0.20
Kissed by an infected person	350 (70)	191 (77)	0.05	122 (66)	46 (68)	0.83
Sat on the lap of an infected person	445 (89)	229 (93)	0.13	175 (95)	66 (97)	0.45
Spent <2 h in the room with an infected person	46 (9)	5 (2)	0.001	16 (9)	5 (7)	0.71

^1^ Seropositivity was defined by IgG or IgM against viral spike or nucleocapsid protein. Values are in no. (%) unless otherwise indicated. ^†^ Included time unsupervised in a fenced yard or tied up (dogs only) and time off the property.

**Table 3:** Association between SARS-CoV-2 seropositivity and clinical signs in U.S. dogs and cats with household exposure to human COVID-19 cases between 1 January 2020 and 10 March 2022 ^1^.

Clinical Signs	Dogs			Cats		
	Seronegative N = 500	Seropositive N = 247	*p* Value	Seronegative N = 185	Seropositive N = 68	*p* Value
Any clinical sign	67 (13)	28 (11)	0.49	42 (23)	15 (22)	0.87
Respiratory signs ^†^	37 (7)	16 (6)	0.69	35 (19)	10 (15)	0.40
Coughing	25 (5)	9 (4)	0.45	14 (8)	6 (9)	0.74
Sneezing	27 (5)	9 (4)	0.32	30 (16)	8 (12)	0.33
Difficulty breathing	6 (1)	0 (0)	0.98	2 (1)	0 (0)	0.99
Vomiting	14 (3)	3 (1)	0.22	7 (4)	1 (1)	0.41
Diarrhea	13 (3)	3 (1)	0.26	1 (1)	1 (1)	0.50
Decreased appetite	15 (3)	7 (3)	0.93	8 (4)	7 (10)	0.10
Decreased energy	30 (6)	10 (4)	0.31	8 (4)	7 (10)	0.11

^1^ Seropositivity was defined by IgG or IgM against viral spike or nucleocapsid protein. Values are in no. (%) unless otherwise indicated. ^†^ Included coughing, sneezing, and difficulty breathing.

## Data Availability

De-identified data may be made available to researchers who submit a methodologically sound proposal to the corresponding author.
